# Tongue Postures and Tongue Centers: A Study of Acoustic-Articulatory Correspondences Across Different Head Angles

**DOI:** 10.3389/fpsyg.2021.768754

**Published:** 2022-01-17

**Authors:** Chenhao Chiu, Yining Weng, Bo-wei Chen

**Affiliations:** National Taiwan University, Taipei, Taiwan

**Keywords:** tongue posture, tongue center, head angles, ultrasound, GAMMs

## Abstract

Recent research on body and head positions has shown that postural changes may induce varying degrees of changes on acoustic speech signals and articulatory gestures. While the preservation of formant profiles across different postures is suitably accounted for by the two-tube model and perturbation theory, it remains unclear whether it is resulted from the accommodation of tongue postures. Specifically, whether the tongue accommodates the changes in head angle to maintain the target acoustics is yet to be determined. The present study examines vowel acoustics and their correspondence with the articulatory maneuvers of the tongue, including both tongue postures and movements of the tongue center, across different head angles. The results show that vowel acoustics, including pitch and formants, are largely unaffected by upward or downward tilting of the head. These preserved acoustics may be attributed to the lingual gestures that compensate for the effects of gravity. Our results also reveal that the tongue postures in response to head movements appear to be vowel-dependent, and the tongue center may serve as an underlying drive that covariates with the head angle changes. These results imply a close relationship between vowel acoustics and tongue postures as well as a target-oriented strategy for different head angles.

## Introduction

In most speech scenarios, speakers talk in an upright position with their eyes looking straight ahead. This upright posture creates a turning angle in the upper vocal tract, separating it into two tubes: the lingual (front) and (pharyngo-)laryngeal (back) tubes. However, the angle between the two tubes is subject to change in different speech scenarios. For example, the angle when speaking to someone on the second floor while you are on the ground floor would differ from when speaking to someone across from you as you look down at the phone in your hands. Recent research on body and head positions has shown that postural changes may induce varying degrees of changes in acoustic speech signals (Flory, 2015; Vorperian et al., 2015) and articulatory gestures (Kitamura et al., 2005; Serrurier and Badin, 2008). While the formant profiles across different postures is suitably accounted for by the two-tube model (Stevens, 1998; Fant, 2006) and perturbation theory (Chiba and Kajiyama, 1941), the impact the angle between the front and back tubes has on vowel acoustics has not been empirically examined. Studies on the resonances and wave propagation of curved ducts revealed that the curvature induces complicated changes in resonance frequencies (e.g., Rostafinski, 1976; Cabelli, 1980). Notably, the direction of the frequency shift is frequency dependent (Félix et al., 2012). The acoustics of these curved ducts were largely established through the examination of hard walled materials or rigid ducts which are assumed not to absorb acoustic energy (Cabelli, 1980; Malbéqui et al., 1996). Literature in music has reported that the wall materials of the wind instruments have influence on the quality of produced tones, such as damping or frequency amplification (see Bucur, 2019 for reviews). The human vocal tract, on the other hand, serves as a very special case in which the tubes are composed of soft tissues (i.e., lossy materials that absorb acoustic energy) with a flexible pivot between the front and back tubes. It is yet to be determined whether these factors would introduce any acoustic differences in speech quality.

In constructing a 3D model of the velum and nasopharyngeal wall, Serrurier and Badin (2008) pointed out that tilting the head predominantly impacts the pharyngeal wall, while the vocal and nasal tract may also undergo independent actions in response to the head tilt. Kitamura et al. (2005) used MRI imagining to compare the vocal tract shapes in the upright and supine positions. Their results showed that soft tissues, including the tongue, velum, and lips, are subject to deformation due to gravitational effects. The effects of gravity are also observed not only in tongue shape, position, movements (Tiede et al., 1997, 2000; Stone et al., 2002) but also in muscle activity of the tongue (Niimi et al., 1994) and velum (Moon and Canady, 1995). In addition, gravity also impacts on the acoustics of sustained vowels (e.g., lower formant bandwidth, Tiede et al., 1997). Apart from different body positions (upright vs. supine), head postures also induce systematic acoustical differences. By examining the frequency perturbation (jitter) and amplitude perturbation (shimmer), Lin et al. (2000) reported that head extension is mostly associated with an increase in fundamental frequency and with a decrease in shimmer. Nevertheless, no study has empirically examined whether the tongue accommodates the changes in head angles in order to maintain acoustic targets. Considering the direct relationship between vowel acoustics and articulatory tongue postures, we would expect the tongue to compensate for different head angles (i.e., the angles between the two tubes).

Physiologically, the tongue is a muscular hydrostat, just like a water balloon. If you squeeze one part of a hydrostat, the fixed volume is displaced, causing other parts to bulge. Previous studies have summarized three major patterns of tongue movements: pivotal rotation, arching/de-arching, and shift (Iskarous, 2005; Kim et al., 2019). As the tongue is composed of intrinsic and extrinsic muscles, controlling tongue positions and postures requires precise fine motor control. In principle, the degree of freedom of the tongue is unlimited. If the degrees of freedom are not significantly reduced, a speaker would struggle to master precise control of the tongue. By reducing the degrees of freedom for a hydrostat like the human tongue, tongue postures can be better controlled and acquired more efficiently (Iskarous, 2005; Gick, 2016; Moisik et al., 2019). The degrees of freedom limiting tongue postures are described in two dimensions: high vs. low and front vs. back. However, these labels describe the tongue’s final position rather than its movement. It should be noted that the reduction of degrees of freedom is associated with the control of a large number of deformable or moving components and with the limited ways of movement, including trajectories and moving mechanism (whether axial or rotational movements). That is to say, if the tongue’s degree of freedom is reduced for speech production, this reduction should occur during movement and posturing, not at the final, intended position.

Hashimoto and Sasaki (1982) was one of the earliest studies to investigate the relationship between tongue shape and tongue position for vowels, treating the tongue surface as a quadratic curve. Using cineradiography, they characterized five parameters for the tongue curve: the horizontal and vertical positions, radius of curvature at the vertex, eccentricity, and the angle of backward lean of the axis of the curve. They used mandible-based rectangular coordinates to characterize the tongue shape and positions relative to the mandible. Their regression analyses revealed that the more the tongue retracts, the more it leans backward, and that the lower the tongue is, the flatter it becomes. Other researchers have proposed a similar approach to fit the tongue through circles (e.g., Iskarous et al., 2003; Stone, 2005). This approach, along with Hashimoto and Sasaki’s (1982) radius of the curvature, suggests that the tongue body center can be mathematically estimated and can thus be treated as a parameter for speech motor control. This concept was later captured in the forward model implemented in CASY (cf. Figure 1 in Lammert et al., 2013). Following that, Parrell et al. (2019) compared how the tongue moves in response to different types of sensory feedback by examining the movement trajectories and end points of the tongue center during the simulation of vowel sequence [, a, i]. Their results reported lower variability of both movement trajectory and end point of the tongue center when somatosensory feedback is available. These literature suggest that the tongue center might be a meaningful parameter in characterizing tongue movements. However, it has yet to be determined if the tongue center displays any consistent movements in speech produced with different speaking postures (e.g., different head angles). If the tongue center behaves consistently across different head angles in different vowel contexts, it would suggest that global stabilization of the tongue is preserved through stable movements of the tongue center.

The current study raises two questions. First, can vowel acoustics be maintained across different head angles, and do tongue postures accommodate these changes accordingly? Second, can the tongue center serve as an underlying force shared by different speech targets (i.e., vowels) in different speech tasks (i.e., speaking with different head angles)? An ultrasound experiment was designed to address these questions.

## Method

### Participants

Eleven native speakers of Taiwan Mandarin (5 female and 6 male; aged 18–24) participated in the experiment. None of them reported any auditory or visual disabilities and were naïve to the purpose of the study. The experiment was conducted in accordance with ethical guidelines approved by National Taiwan University.

### Apparatus

The ultrasonography recordings were collected using a portable ultrasound machine (CGM OPUS5100) with a transvaginal electronic curved array probe (CLA 651). The transducer was fixed at 30 degrees away from the speaker’s chest and was adjusted along the midsagittal tongue contour. A Samson C01U hyper-cardioid condenser microphone was placed directly facing the participant’s mouth approximately 20 cm away. Acoustic and ultrasound data were recorded simultaneously with a USB 3.0 powered capture card (ExtremeCap U3) and saved as .mp3 and .mp4, respectively. Acoustic signals were sampled at 48,000 Hz, and the frame rate for the ultrasound videos was set at 40 fps.

### Procedures and Stimuli

Participants were instructed to sit upright, facing the wall at a distance of 60 cm away. The experimenters then fixed the ultrasound probe to the assigned position with the stabilization headset (Articulate Instrument Inc.). The probe was adjusted to a level that was comfortable for the participant and that would capture images clear enough to identify and trace. The experiment involved repetitions of [i, a, u] in isolation at eight different head angles: −15°, −10°, 0° (H00), +10°, +15°, +45°, +60°, and +90°. The angles were marked on a measuring tape affixed to the wall in front of the participant, and the 0° baseline was adjusted for each participant’s height. At each angle, participants were instructed to produce the same vowel ten times consecutively. Each vowel was sustained for roughly 1 second, with another second of interval between vowel production. A total of 240 trials (3 vowels × 8 angles × 10 tokens) were collected for each participant.

### Data Preparation and Analyses

#### Acoustics

The vowel boundaries were first labeled in Praat for *F*0 and formant analyses. *F*0, *F*1, and *F*2 values were obtained from the midpoint of the labeled vowel interval, using the built-in functions in Praat. Pitch and formant data were normalized (in time),^1^ standardized (in z-score),^2^ and then fitted into generalized additive mixed models (GAMMs; Wieling, 2018) with 95% confidence intervals around the predicted fit. The full model formula for *F*0 in the [a] context is provided in Eq. 1. The same formula was also applied to the other vowels and formants.


(1) bam(z.F0∼Angle+s(norm.Time,bs=c″s″,

k=10)+s(norm.Time,bs=c″s″,k=10,

by=Angle)+te(norm.Time,Trial,by=Angle)

+s(norm.Time,Subject,bs=f″s″,k=10,

m=1,by=Angle),discrete=TRUE,

method=f″REML″,data,rho,AR.start)3

#### Tongue Postures

Ultrasound images of tongue postures were captured from the midpoint of the labeled vowel interval, using a customized MatLab script. The postures were then manually traced using a livewire algorithm in MatLab. The tongue traces were converted into polar coordinates, since polar coordinates allow one to compare tongue traces roughly perpendicular to the tongue surface and to reduce estimation errors at the tongue tip and root (Heyne and Derrick, 2015; Mielke, 2015). For polar coordinate conversion we closely followed Heyne and Derrick (2015): we first estimated the virtual origin coordinates (*X*_*O*_, *Y*_*O*_) of our radial ultrasound transducer; the series of points that define a tongue trace could then be converted to polar coordinates by taking the virtual origin as the polar center; points in the form of (*x*_*i*_, *y*_*i*_) were converted to (θ_*i*_, *r*_*i*_), where θ_*i*_ is calculated from atan($\frac{\left(y_{i}-Y_{O}\right)}{\left(x_{i}-X_{O}\right)}$), and *r*_*i*_ is the Euclidean distance between a point and the virtual origin. The converted polar tongue traces were then rotated by 30° to correct for the probe placement and fitted into GAMMs with 95% confidence intervals around the predicted fit. To fit predicted tongue contours, a virtual origin was used as the polar origin, following the method introduced in Heyne and Derrick (2015). The full model formula for tongue shape contour in the context of vowel [a] is provided in Eq. 2. Separate models with the same formula were constructed for the other two vowels.


(2) bam(Y∼s(X,bs=t″p″,k=40)+s(X,

Participant,bs=f″s″,m=1),

method=M″L″,data,rho,AR.start)

#### Tongue Centers

Tongue centers were estimated through circle-fitting using the traced tongue contours. Circle-fitting was based on a simple least squares method that minimized the sum of squared deviations of a given tongue contour from a fitted circle, using the optim() function in R. The function identified the optimal radius and center coordinates of a fitted circle such that its sum of squared deviations was the lowest. Specifically, the sum of squared deviations *SS* was defined as Eq. 3:^4^


(3) $$S\,S=\sum_{i=1}^{n}{\left(\sqrt{{\left(x_{i}-X_{c}\right)}^{2}+{\left(y_{i}-Y_{c}\right)}^{2}}-r\right)}^{2}$$

where (*x*_*i*_, *y*_*i*_) = coordinates of a series of *n* points that define a tongue contour

(*X*_*c*_, *Y*_*c*_) = center of the fitted circle

*r* = radius of the fitted circle

Additionally, we constrained the radii of best-fit circles for tongue contours that were not taken at 0° (H00): for the 8 contours (8 angles) that had the same token number, the best-fit radius and center coordinates for the contour at H00 was first calculated; then H00’s best-fit radius was set as a fixed input parameter for other angles’ circle-fitting procedure, so that only the best-fit centers would be obtained for the remaining 7 angles’ contours. The “method” argument in the optim() function was specified as “L-BFGS-B” because the default method produced erroneous results when the radius was constrained in some cases.

The obtained tongue centers at each angle were first aggregated by participant, then averaged across different participants. Mean square errors (MSE) of each fitted circle (i.e., individual trials) were aggregated by vowel.

## Results

### Acoustics

**FIGURE 1:**
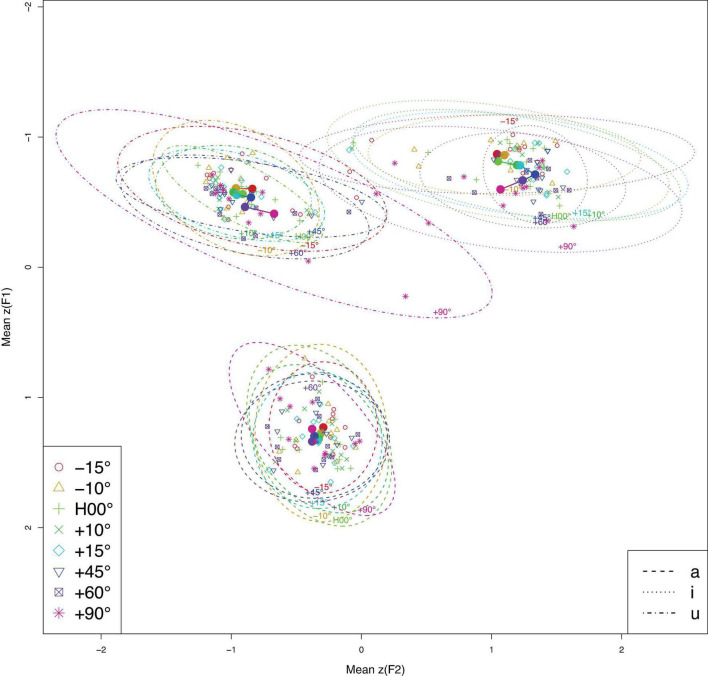
Averaged formants (*F*1∼*F*2, in *z*-score) across different head angels (color-coded) for all three vowels (line type separated). Hollow circles represent the averaged formants for individual speakers; solid circles represent the averaged formants across all speakers at each head angle. Ellipses enclose 95% of the normal probability density function (color online).

Acoustically, *F*0 and the two measured formants (*F*1 and *F*2) remained largely unaffected across the different head angles for all three vowels as depicted by the overlapped fitted contours in the GAMMs results (see Supplementary Figures for details), though the *F*1 trajectory at −10° appeared to be more erratic than the *F*1 trajectories at other angles during the production of [u]. Nevertheless, this fluctuation did not result in any statistical differences in any pair-wise comparison. The distribution of averaged formants across different head angles is presented in Figure 1. Overall, the results of all three vowels are indicative of acoustic preservation across the different head angles.

### Tongue Postures

**FIGURE 2:**
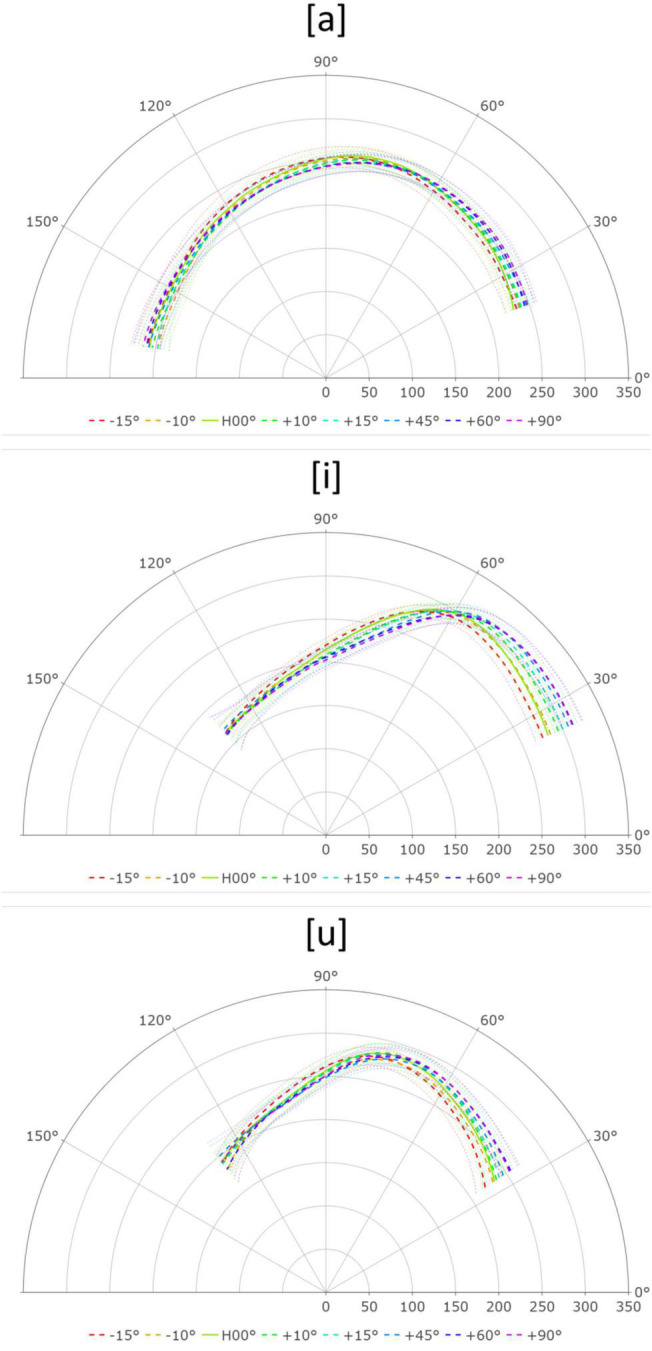
Tongue surface contour for [a], [i], and [u] across the eight head angles. Tongue tip is on the right.

Articulatorily, the tongue is lowered and retracted toward the pharyngeal wall in the production of [a]. As the angle of the head increased (head-up position), gravity pulled the tongue root toward the pharyngeal wall. Thus, less force was required to achieve the intended tongue root position. This interpretation is supported by the reduced depression of the tongue tip at higher head angles (Figure 2 top). The GAMM pair-wise comparison showed that the tongue posture did not change significantly across different head angles (see Supplementary Figures).

In terms of the tongue posture for [i] across different head angles, it was observed that as the head angle increased, the front of the tongue continued to rise while the dorsum of the tongue was lowered, thus creating a pivotal rotation for the tongue posture (Figure 2 center). The pivotal point was roughly fixed on the constriction point of [i]. As the head tilts upward, the tongue root must exert more force to fight against gravity while the tongue tip can remain more relaxed. In contrast, when the head is tilted downward, the gravitational pull squeezes the tongue tip; the tongue root, on the other hand, is rather relaxed.

The tongue posture results for [u] were similar to those for [i]. As the head was lifted, the effect of gravity helped maintain the shape of the tongue tip, reducing the need for muscle contraction. On the other hand, tilting the head downward caused the tongue tip to be pulled down by gravity. Maintaining the tongue shape, therefore, required more effort, and the tongue tip was more compressed. Unlike the [i] results, the positions of the tongue dorsum were not affected by the different head angles (Figure 2 bottom). We suspect this is because the constriction for [u] is further back in the oral cavity, leaving the tongue dorsum fewer degrees of freedom to move around.

### Tongue Centers

**FIGURE 3:**
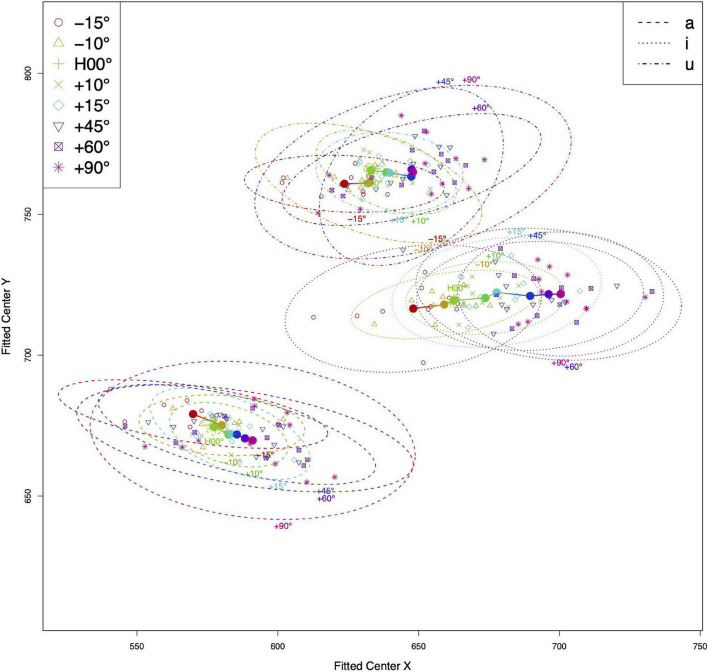
Averaged tongue centers across different head angles (color-coded) for all three vowels (line type separated). Hollow circles represent the averaged tongue center for individual speakers; solid circles represent the averaged tongue centers across all speakers at each head angle. Ellipses enclose 95% of the normal probability density function (color online).

Figure 3 presents the distribution of tongue centers across different head angles. The tongue centers of individual speakers (represented by individual marks in the figure) at each head angle were averaged and then plotted as solid circles. A 95% confidence ellipse encloses the distribution of speaker variance. From the results, we observe two major findings. First and foremost, the tongue centers moved unidirectionally and almost linearly as the head angle increased. Tilting the head upward caused the tongue center to move anteriorly. This pattern was observed for all three vowels, with only a few outliers (i.e., −10° > 0° in [u] and [a]). Despite these outliers, the anterior moving pattern was consistently observed both when the head was tilted down (from −15° to −10°) and up (from 0° all the way to 90°). Second, the distributions of the tongue centers also faithfully reflect the tongue positions for each vowel, and the distributions for each vowel were distinct. The tongue centers occupied the low and back of the upper vocal tract for [a], high and back for [u], and mid and front for [i]. The tongue centers for [i] were scattered around the mid level, as opposed to the high level, possibly because the tongue posture of [i] is more curved. Higher degrees of curving for the [i] postures were validated by higher MSE values for [i] (MSE = 159.65 pixel^2^), compared with the other two vowels (66.73 and 73.54 for [a] and [u], respectively).

## Discussion

The present study investigated how the tongue is postured and centered at different head angles and whether the corresponding acoustics is affected accordingly. Our results showed that pitch and formants are largely preserved across different head angles. The ultrasound results, on the other hand, showed that the tongue postures involved vowel-dependent movements across different head angles. Only limited tongue movements were observed in the [a] context; the tongue underwent a pivotal rotation across different head angles in the [i] and [u] contexts, but to a lesser degree for the tongue dorsum after the constriction point for [u]. Crucially, these results suggest that gravity has an effect on tongue postures, especially for the high vowels [i] and [u]. We are seldom conscious of this gravitational effect because we typically speak in an upright stance. Once the head posture changes, the gravitational effects emerge. As revealed in our results, for different vowels, different parts of the tongue fight against gravity to achieve the intended positional target and maintain the intended acoustics. Similar postural compensations were also observed in Kitamura et al. (2005), though they did not report acoustic data. Our results show that articulatory targets in terms of lingual constrictions are achieved in order to preserve the intended acoustics leading us to conclude that the tongue employs a target-oriented strategy when the angles between the front and back tubes change due to head tilting.

**FIGURE A2:**
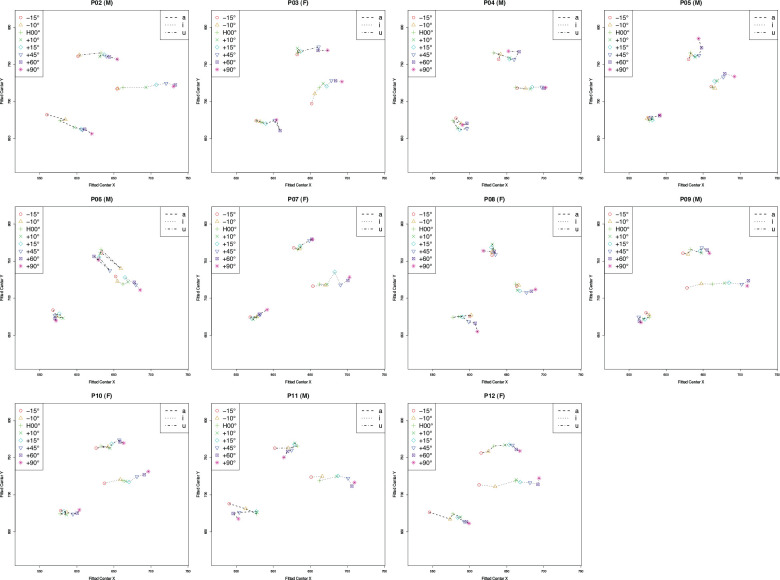
Tongue center trajectories across different head angles arranged by participant. Trajectory patterns do not seem to differ systematically based on gender (male vs female).

As for the tongue center, our results showed that the estimated tongue centers moved collinearly with the head angle. While some individual variations were observed, these variations did not form any consistent patterns. Tongue center trajectories of each participant are included in the appendix for further reference (Figure A2). The positive correlation of anterior movement of the tongue center and increase in head angle also confirmed the view that the tongue is postured and centered in such a way to overcome gravity in order to achieve the intended articulatory and/or acoustic targets. Crucially, the uniform movement of the tongue center across different vowels and tokens also suggests that the tongue center may act as an underlying control parameter in tongue movements for speech. However, the tongue may be postured, the global stabilization of the tongue is secured through the steady movement of such a center.

Using articulatory trajectory data such as EMA, some studies have recently constructed articulatory animation systems to simulate emotional speech (e.g., Yu et al., 2017) and even to serve as visual biofeedback (e.g., Fabre et al., 2017). Our findings in the current study could potentially provide additional implementations to those animation systems (e.g., Wang et al., 2012; Yu et al., 2017) as well as neural network architecture (cf. Yu et al., 2019). Further examinations would be required.

Finally, we acknowledge two limitations of the current study. First, gravity also affects the ultrasound probe and stabilizer. It is yet to be determined how much this contributes to the final results. We acknowledge the need to correct for the decoupled movements between the jaw and tongue, using optical tracking systems to analyze the relative positioning of the jaw, stabilizer, and the probe (e.g., HOCUS in Whalen et al., 2005). Second, the stabilizer confines jaw movement, potentially affecting the articulation of vowels that requires significant jaw lowering, especially at higher head angles. These factors warrant further examination. Additionally, it is worth investigating the dynamic changes involved throughout vowel production at different head angles.

## Data Availability Statement

The original contributions presented in the study are included in the article/Supplementary Material, further inquiries can be directed to the corresponding author.

## Ethics Statement

The studies involving human participants were reviewed and approved by Research Ethics Committee of National Taiwan University. The patients/participants provided their written informed consent to participate in this study.

## Author Contributions

CC and YW contributed to conception and design of the study and the selection of statistical analyses. YW was responsible for the statistical analyses and figure generation, took charge of the experiment set up, and conducted the experiment with B-WC. B-WC performed the tongue tracing for all the data. CC wrote the first draft of the manuscript. All authors contributed to manuscript revision, read, and approved the submitted version.

## Conflict of Interest

The authors declare that the research was conducted in the absence of any commercial or financial relationships that could be construed as a potential conflict of interest.

## Publisher’s Note

All claims expressed in this article are solely those of the authors and do not necessarily represent those of their affiliated organizations, or those of the publisher, the editors and the reviewers. Any product that may be evaluated in this article, or claim that may be made by its manufacturer, is not guaranteed or endorsed by the publisher.

## Figures and Tables

**FIGURE A1:**
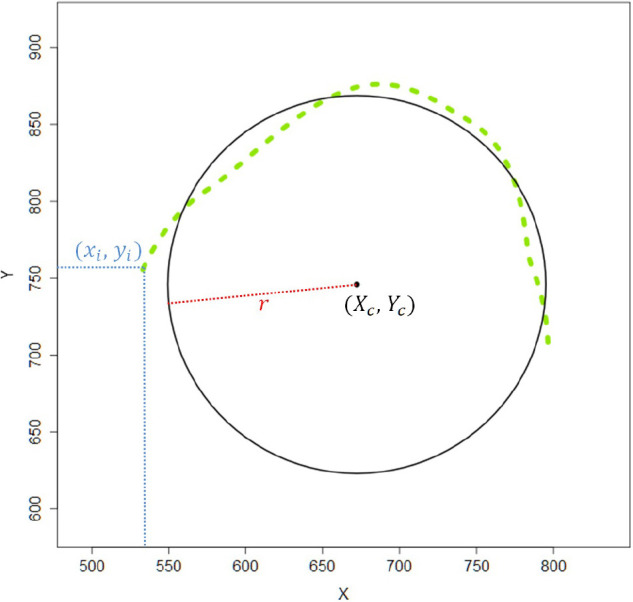
Illustration for tongue center estimation as defined in Eq. 3. Dashed curvy green line is an example tongue contour. Dotted straight blue lines locate the first point of the tongue contour. Solid black circle is the best-fit circle calculated from the given tongue contour. Estimated center and radius of the best-fit circle are also displayed (color online).

## Footnotes

1 The formula for time normalization was: x_norm_ = [x - min(x)]/[max(x) - min(x)].

2 The formula for *z*-score transformation was: *z*.*F*0 = [*F*0-mean(*F*0)]/sd(*F*0).

4 An illustrative figure depicting the estimation of tongue center is included in the appendix (Figure A1).
