# Supplementary figures and images for: Tongue Postures and Tongue Centers: A Study of Acoustic-Articulatory Correspondences Across Different Head Angles
Source: Front Psychol. 2022 Jan 17;12:768754. doi: 10.3389/fpsyg.2021.768754 (PMC8801537; doi:10.3389/fpsyg.2021.768754)

Vowel /a/, pairwise comparisons

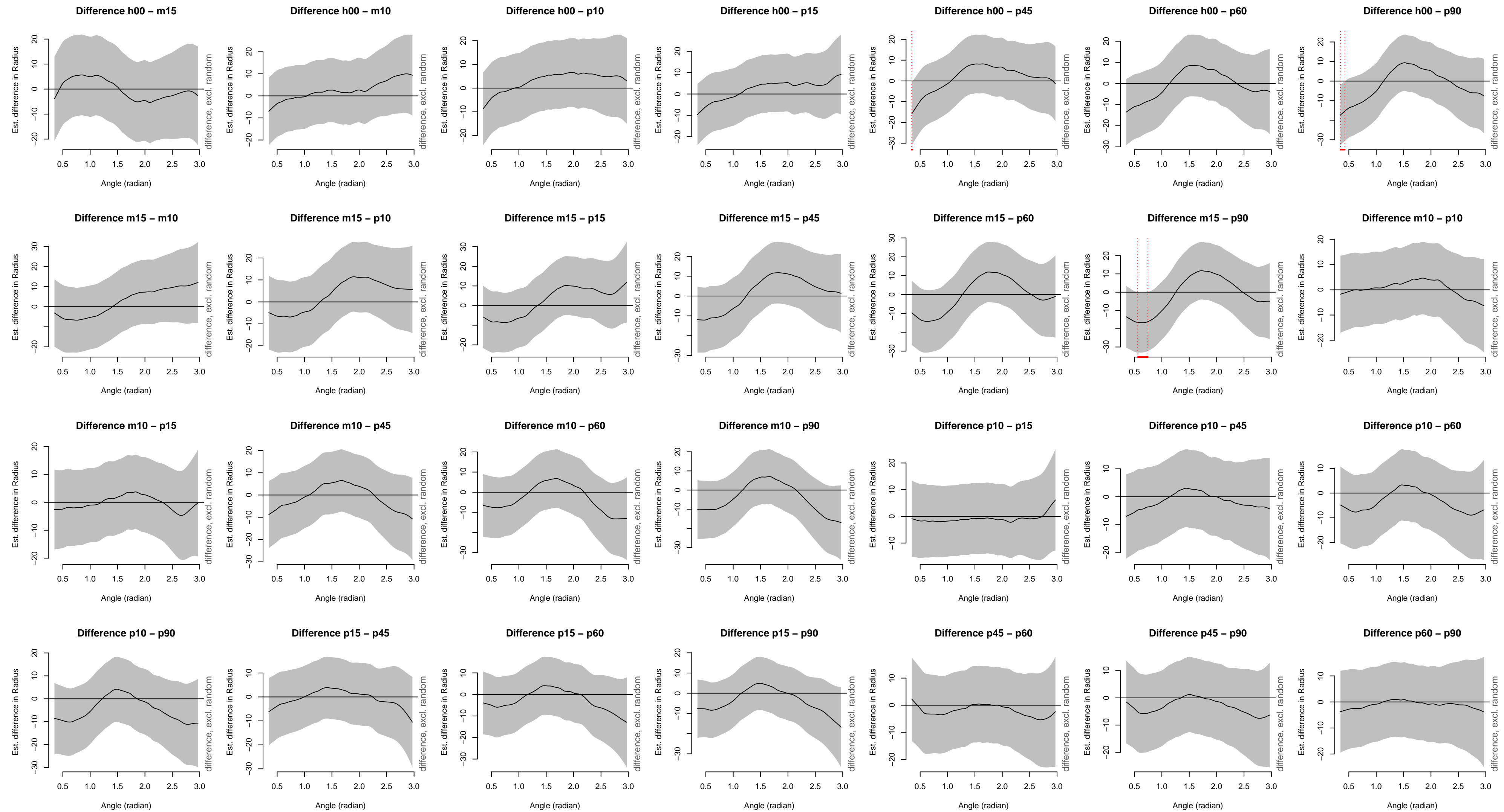

Supplement: Supplementary file 1 [file Data_Sheet_1.PDF]

Vowel /i/, pairwise comparisons

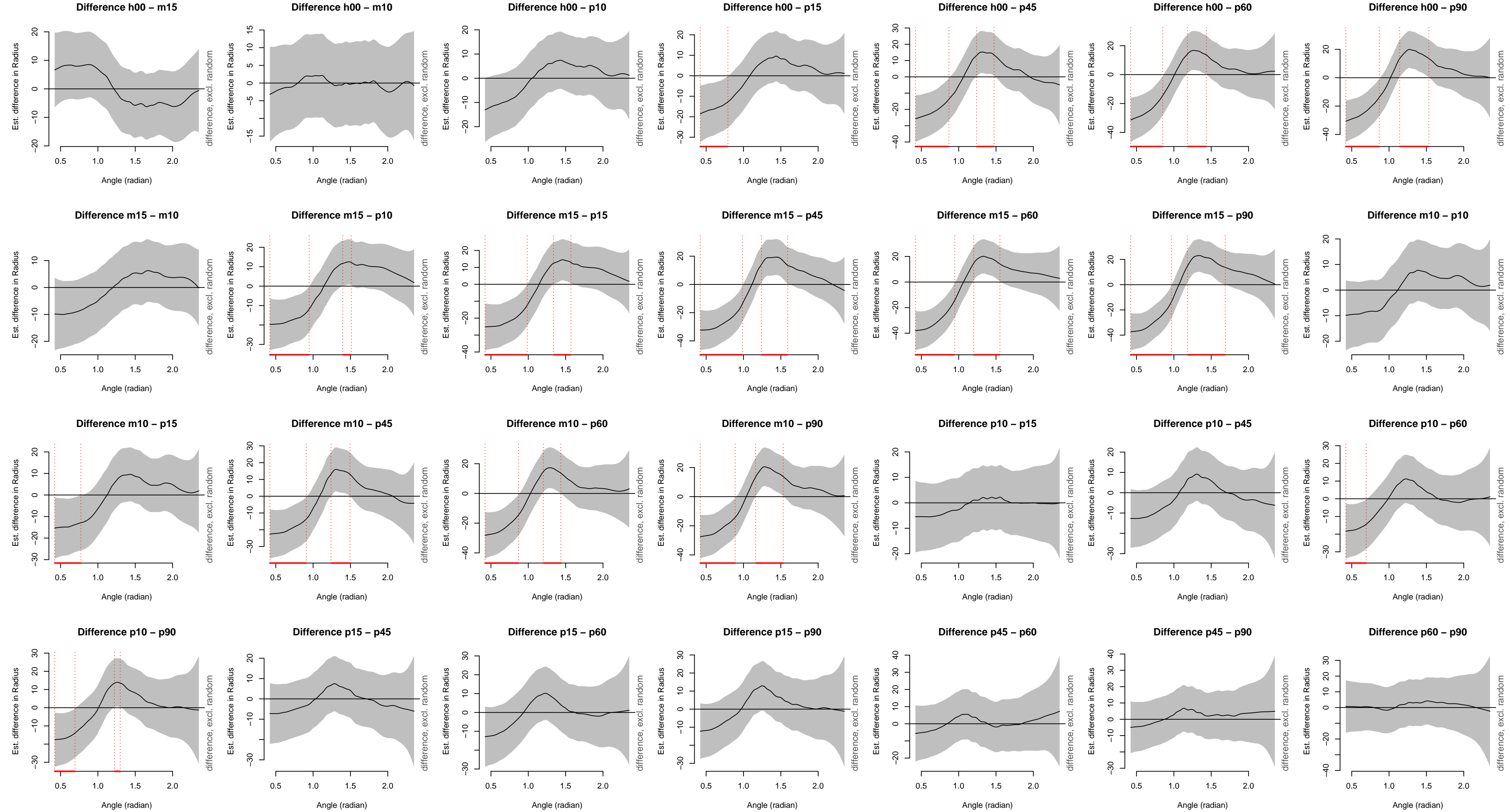

Supplement: Supplementary file 2 [file Data_Sheet_2.PDF]

Vowel /u/, pairwise comparisons

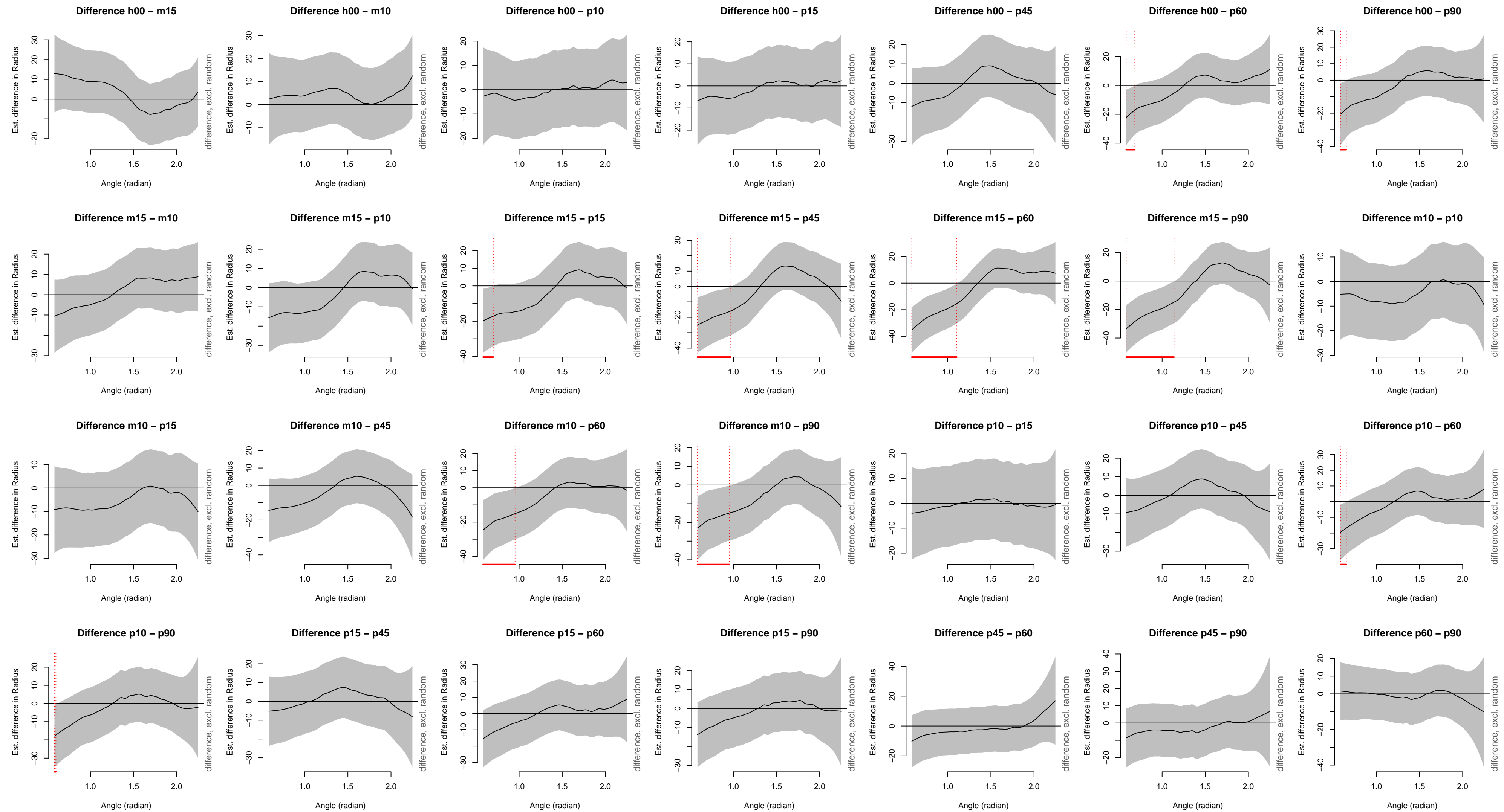

Supplement: Supplementary file 3 [file Data_Sheet_3.PDF]

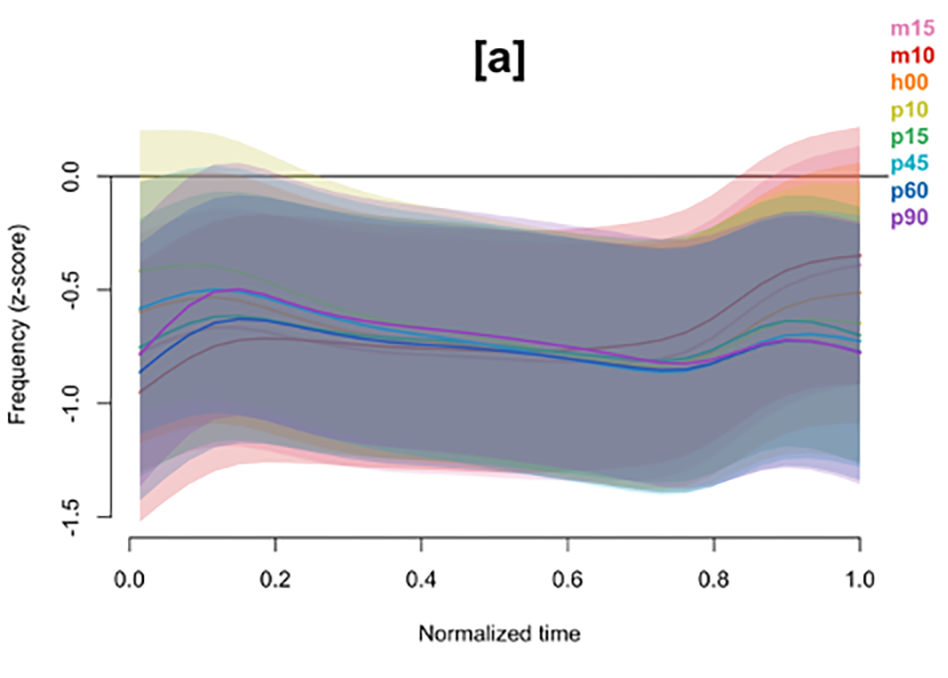

Supplement: Supplementary file 4 [file Image_1.PNG]

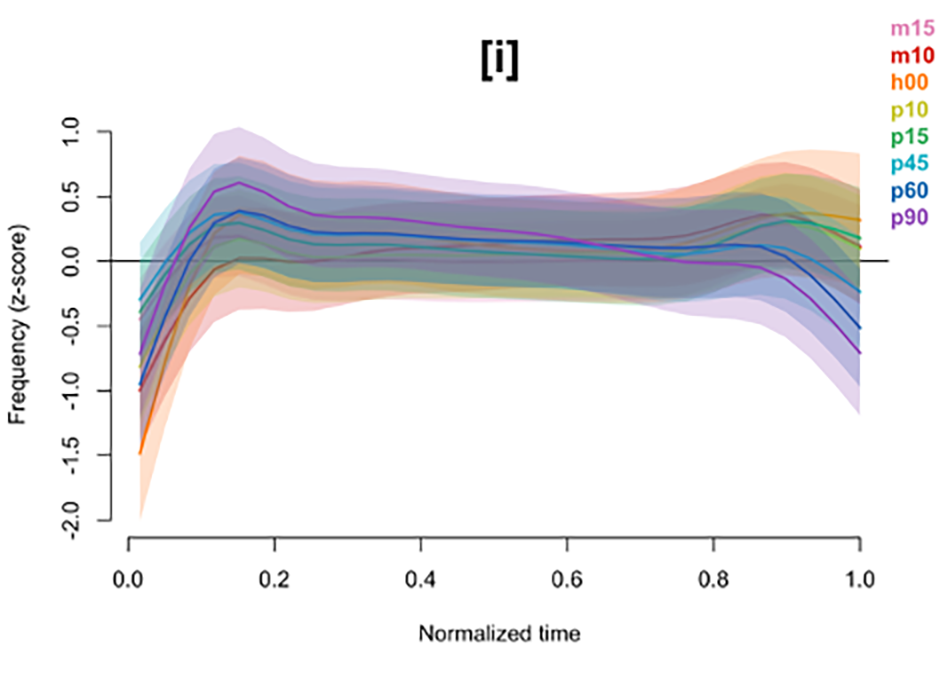

Supplement: Supplementary file 5 [file Image_2.PNG]

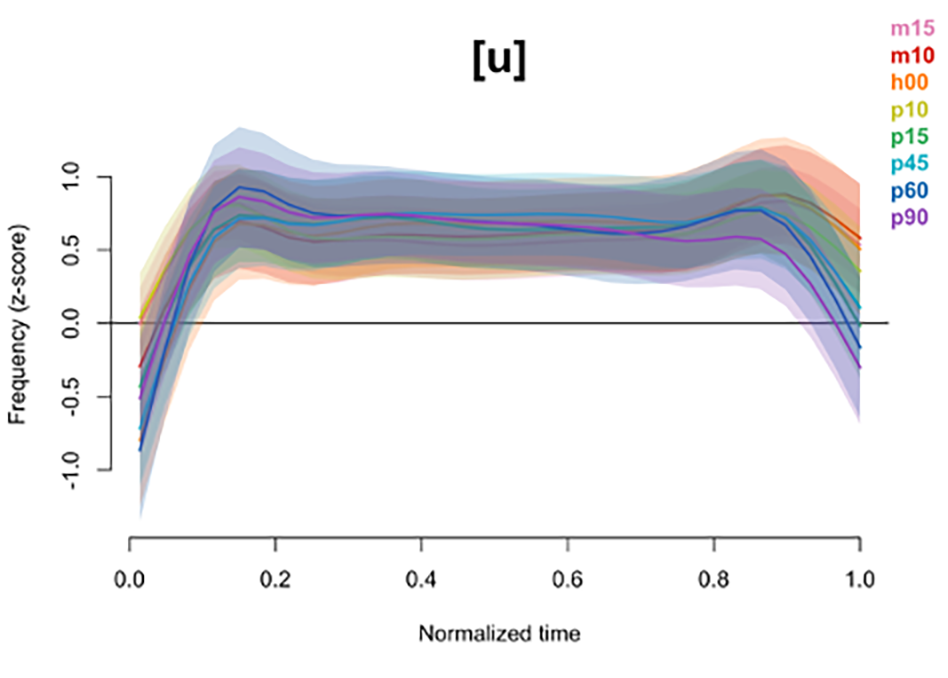

Supplement: Supplementary file 6 [file Image_3.PNG]

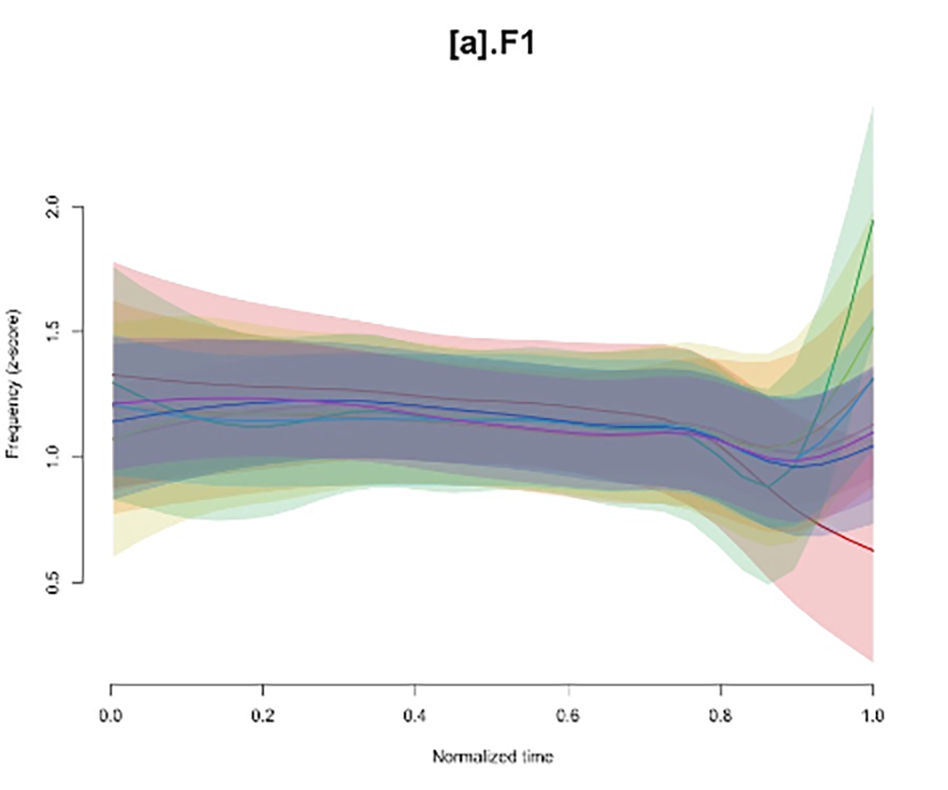

Supplement: Supplementary file 7 [file Image_4.PNG]

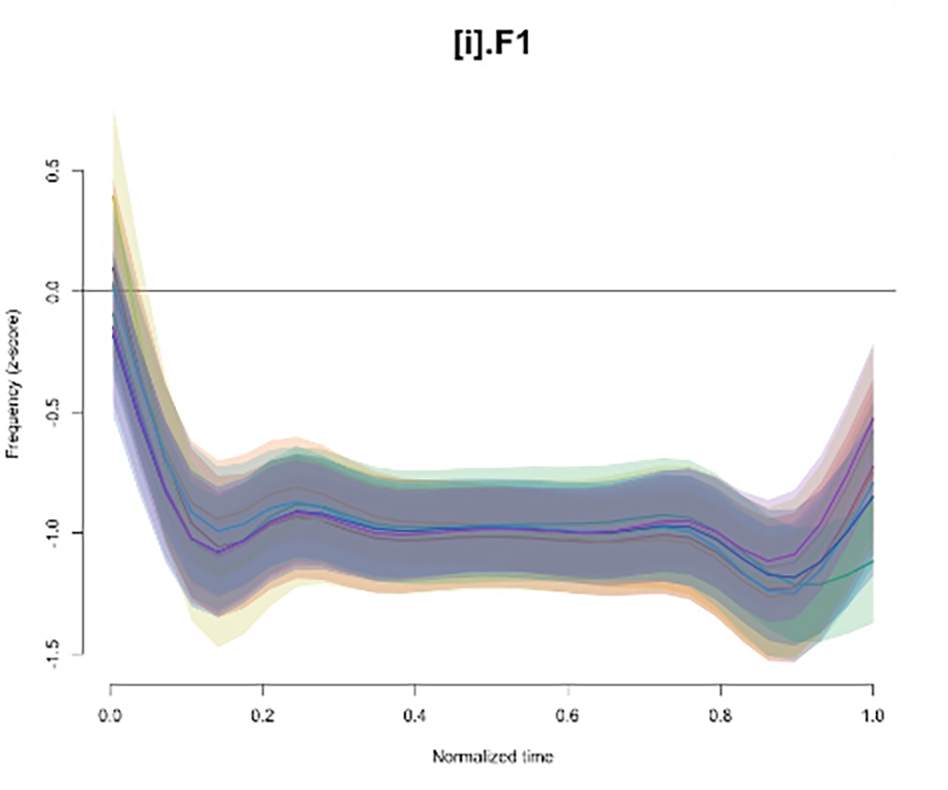

Supplement: Supplementary file 8 [file Image_5.PNG]

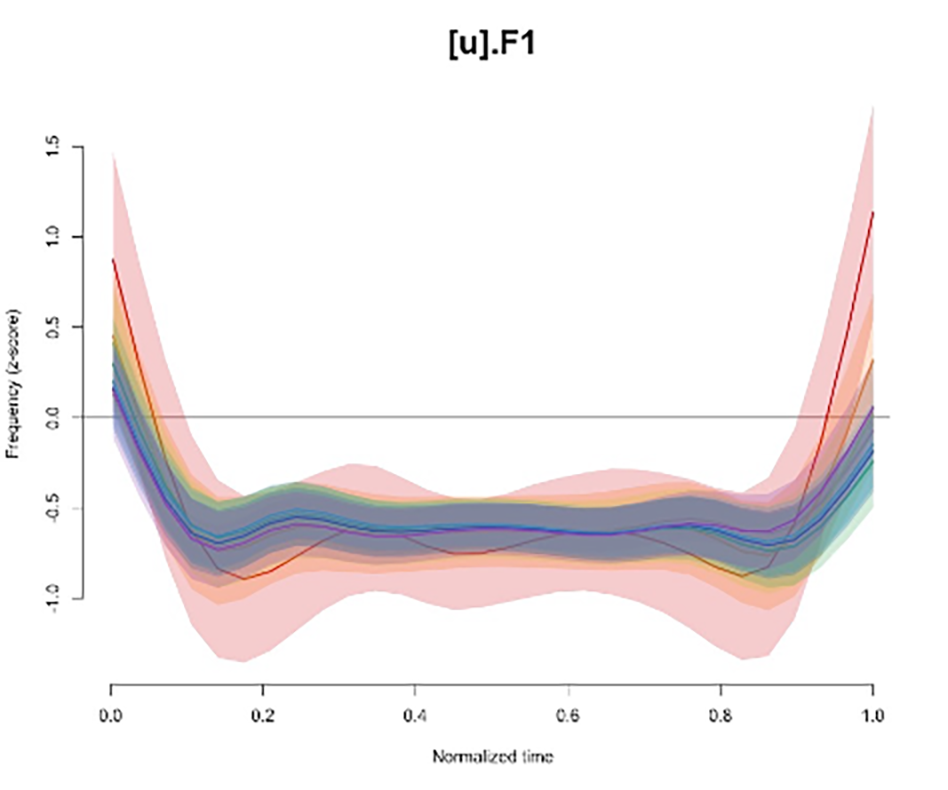

Supplement: Supplementary file 9 [file Image_6.PNG]

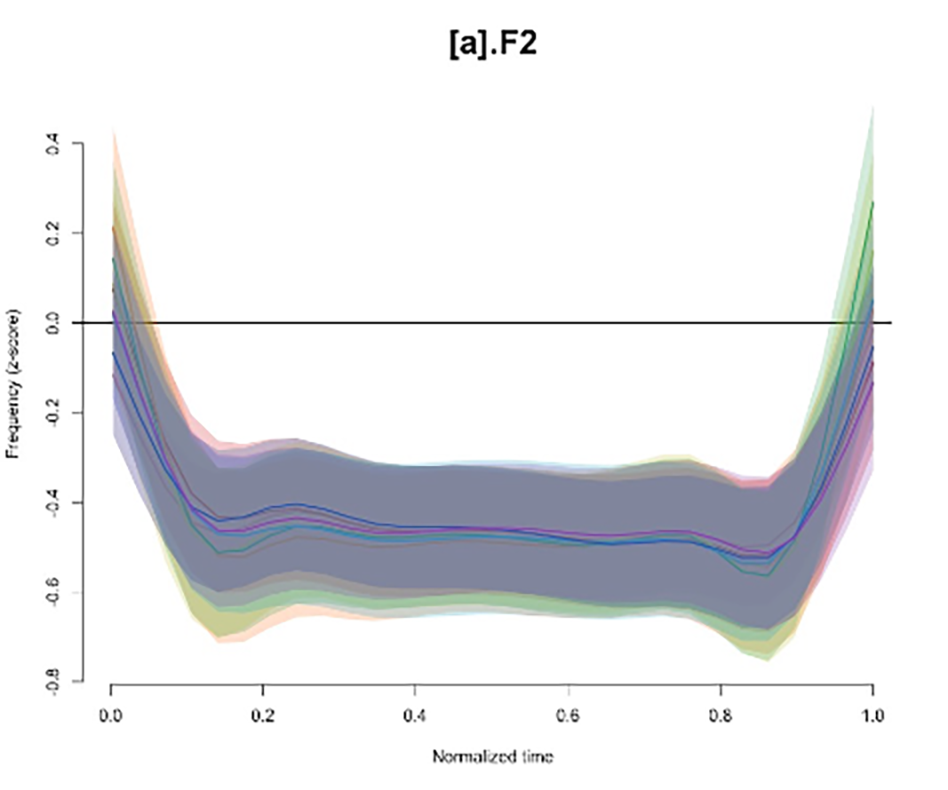

Supplement: Supplementary file 10 [file Image_7.PNG]

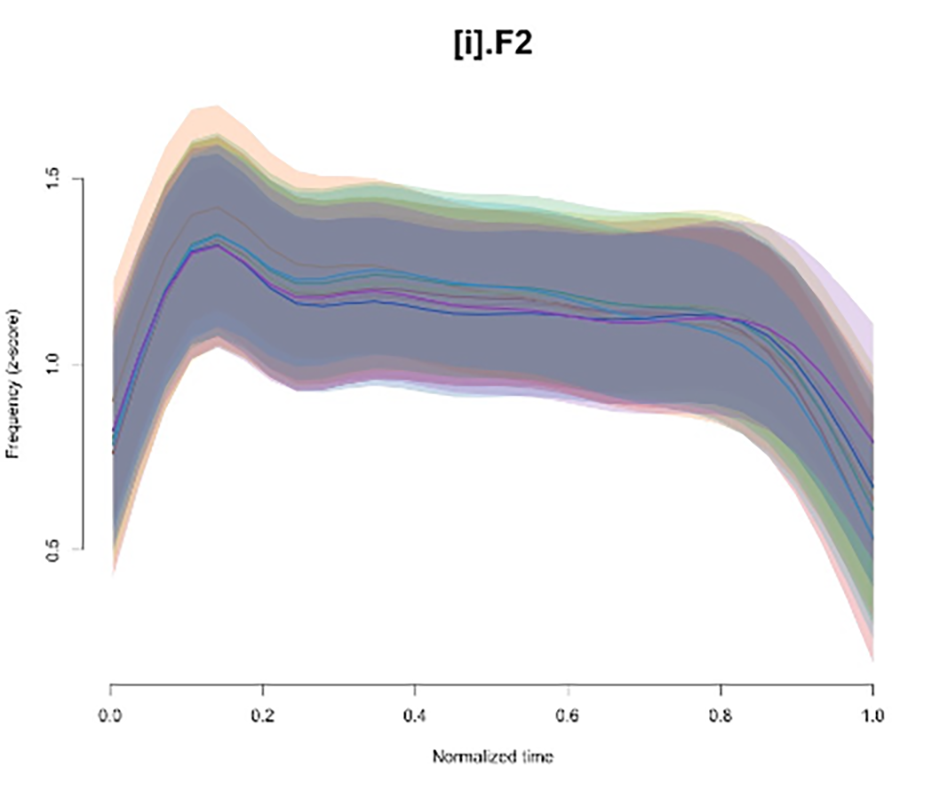

Supplement: Supplementary file 11 [file Image_8.PNG]

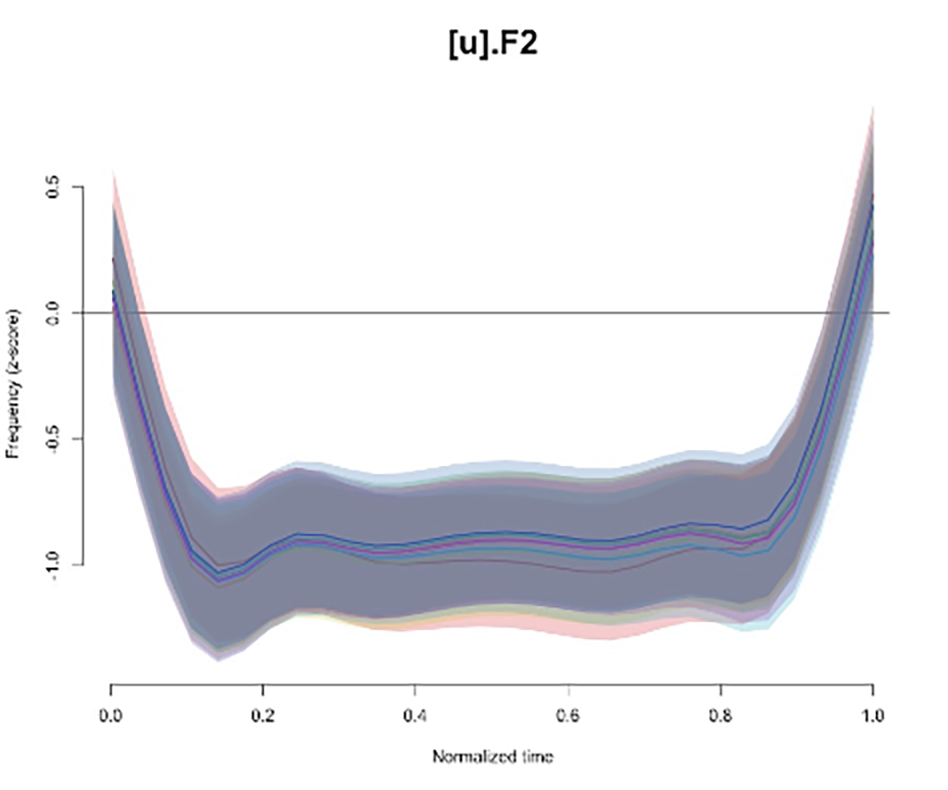

Supplement: Supplementary file 12 [file Image_9.PNG]
